# Emerging Roles of Biomolecular Condensates in Pre‐mRNA 3′ End Processing

**DOI:** 10.1002/wrna.70024

**Published:** 2025-08-13

**Authors:** Yoseop Yoon, Liang Liu, Cailyx Quan, Yongsheng Shi

**Affiliations:** ^1^ Department of Microbiology and Molecular Genetics School of Medicine, University of California, Irvine Irvine California USA

**Keywords:** biomolecular condensate, membraneless organelle, mRNA 3′ processing, nuclear speckle, polyadenylation

## Abstract

Biomolecular condensates are membraneless assemblies of proteins and nucleic acids, often formed through liquid–liquid phase separation. They selectively concentrate specific biomolecules and play essential roles in diverse cellular processes and diseases. This review discusses the emerging roles of biomolecular condensates in pre‐mRNA 3′ end processing, a critical step in mRNA biogenesis. 3′ end processing factors are enriched in intrinsically disordered regions and undergo phase separation to form condensates that, in turn, fine‐tune the efficiency and specificity of 3′ end processing. Additionally, we describe how distinct 3′ end processing pathways are spatially and functionally compartmentalized within nuclear biomolecular condensates, such as nuclear speckles and histone locus bodies. Finally, we propose that 3′ end processing represents a promising experimental system to investigate fundamental principles underlying biomolecular condensate formation and function.

This article is categorized under:

RNA Interactions with Proteins and Other Molecules > Protein‐RNA Recognition
RNA Interactions with Proteins and Other Molecules > RNA‐Protein Complexes
RNA Interactions with Proteins and Other Molecules > Protein‐RNA Interactions: Functional Implications

## Graphical abstract

Pre‐mRNA 3′ end processing factors can self‐assemble into biomolecular condensates that fine‐tune pre‐mRNA cleavage and polyadenylation, and their misregulation underlies disease. Nuclear speckles and histone locus bodies spatially compartmentalize distinct 3′ end reactions to ensure efficient gene expression control.

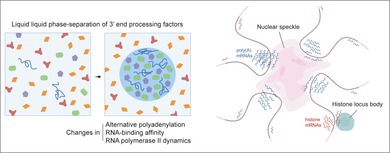

## 1 Introduction

Eukaryotic gene expression relies on precisely coordinated steps—including transcription, splicing, and 3′ end processing—to generate functional mRNAs. Pre‐mRNA 3′ end processing proceeds by two distinct mechanisms: for the vast majority of protein‐coding transcripts, an endonucleolytic cleavage is immediately followed by polyadenylated (poly(A)) tail addition (Chan et al. 2011; Colgan and Manley 1997). By contrast, replication‐dependent histone mRNAs in most metazoans undergo a single cleavage event that leaves a stem‐loop and no poly(A) tail, making them unique among eukaryotic mRNAs (Dominski and Marzluff 2007). In canonical poly(A) mRNAs, the 3′ end cleavage releases the nascent transcript from RNA polymerase II (RNAPII), and subsequent polyadenylation enhances mRNA stability, nuclear export, and translation efficiency (Tian and Manley 2017). Defects in cleavage and polyadenylation disrupt mRNA maturation and transcription termination, potentially causing transcriptional readthrough and interference with downstream genes (Proudfoot 2016).

**FIGURE 1:**
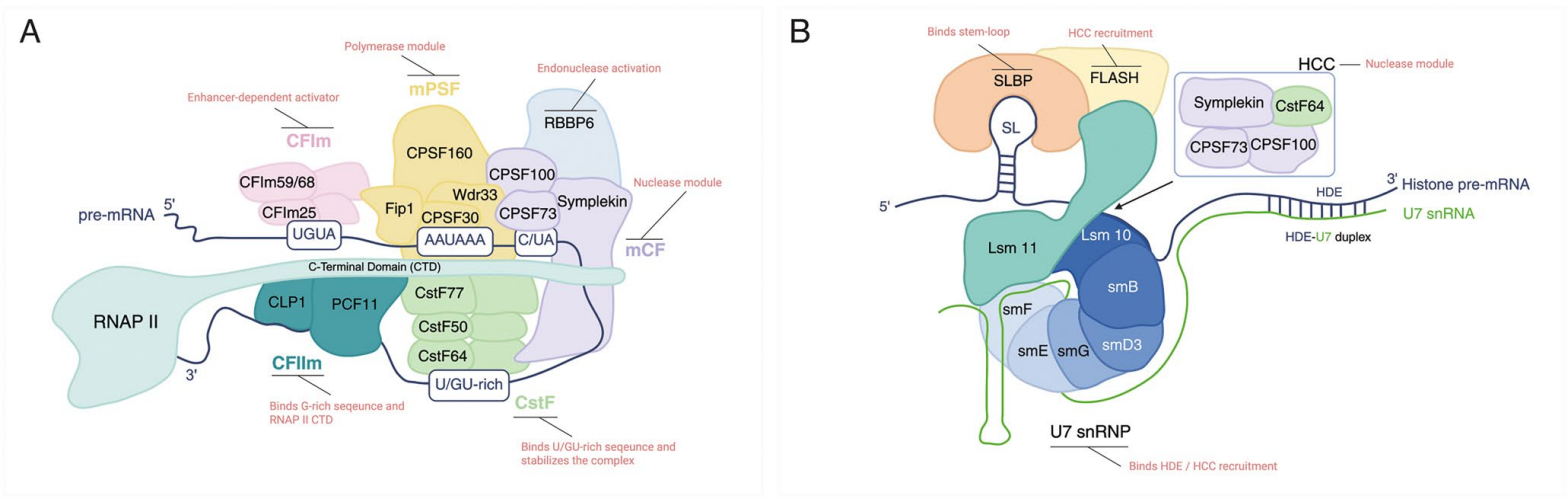
Schematic representation of the 3′ end processing machinery for polyadenylated and histone mRNAs. (A) Processing machinery for polyadenylated mRNAs. mPSF, mammalian polyadenylation specificity factor; mCF, mammalian cleavage factor; CstF, cleavage stimulation factor; CFIm, cleavage factor Im; CFIIm, cleavage factor IIm; RNAP II, RNA polymerase II. (B) Processing machinery for replication‐dependent histone mRNAs (redrawn based on Sun et al. 2020). HCC, histone cleavage complex; HDE, histone downstream element; SL, stem‐loop. In both pathways, the cleavage reaction is catalyzed by the endonuclease CPSF73.

Over the past four decades, the biochemical processes underlying 3′ end processing have been extensively studied for both poly(A) and histone mRNAs. The core machinery for poly(A) mRNA processing includes multi‐protein complexes such as Cleavage and Polyadenylation Specificity Factor (CPSF), Cleavage Stimulation Factor (CstF), Cleavage Factor Im (CFIm), and Cleavage Factor IIm (CFIIm), along with individual proteins such as Retinoblastoma‐Binding Protein 6 (RBBP6) and PolyA Polymerase (PAP) (Figure 1A) (Boreikaite and Passmore 2023; Chan et al. 2011). These factors recognize specific RNA elements, including the polyadenylation signal (PAS)—typically containing an A(A/U)UAAA motif—and auxiliary upstream/downstream sequences. The histone 3′ end processing machinery shares several subunits with CPSF and CstF but also includes unique factors, such as stem‐loop binding protein (SLBP), FLASH, and the U7 snRNP (Dominski and Marzluff 2007) (Figure 1B). Instead of PAS, this machinery recognizes a conserved stem‐loop structure and a histone downstream element (HDE). Recent studies have reconstituted these reactions in vitro, identifying the minimal protein sets required for each process (Boreikaite et al. 2022; Schmidt et al. 2022; Sun et al. 2020).

High‐throughput sequencing methods have revealed another layer of complexity in 3′ end processing over the past ~15 years. Genes across diverse organisms (animals, plants, and fungi) frequently contain multiple PASs (Derti et al. 2012; Jan et al. 2011; Liu et al. 2017; Sanfilippo et al. 2017; Shepard et al. 2011; Smibert et al. 2012; Wu et al. 2011). Differential usage of these sites generates mRNA isoforms with distinct 3′ ends, a phenomenon known as alternative polyadenylation (APA). These isoforms differ in 3′ untranslated regions (UTRs) or coding sequences, influencing mRNA stability, translation efficiency, nuclear export, and subcellular localization (Tian and Manley 2017; Yoon et al. 2019). Dysregulation of APA contributes to diseases such as cancer and neurological disorders (Gruber and Zavolan 2019), and viruses exploit this process to suppress host gene expression (Vijayakumar et al. 2022). The regulation and functional consequences of APA have been extensively reviewed elsewhere (Boreikaite and Passmore 2023; Gruber and Zavolan 2019; Gruber et al. 2014; Mitschka and Mayr 2022; Shi 2012; Tian and Manley 2017).

**FIGURE 2:**
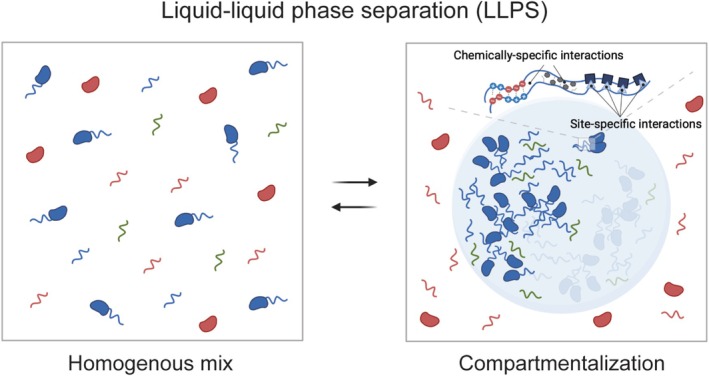
Liquid–liquid phase separation (LLPS) underlies biomolecular condensate formation. LLPS is a biophysical process in which macromolecules self‐organize into distinct, liquid‐like droplets by demixing from the surrounding soluble environment. This process typically involves multivalent interactions among intrinsically disordered protein regions (IDRs), structured protein domains, and nucleic acids, and is concentration‐dependent. Biomolecular condensates dynamically rearrange their internal biomolecules and exchange components with the surrounding environment, selectively compartmentalizing specific biomolecules while excluding others.

Although the biochemical mechanisms of 3′ end processing and APA are well established, recent insights into biomolecular condensates provide a new framework for understanding their spatial and regulatory complexity. Cellular compartmentalization is crucial for organizing biochemical processes, concentrating specific biomolecules to promote reactions, and preventing unwanted interactions. This is achieved through membrane‐bound organelles and, as is increasingly appreciated, through membrane‐less biomolecular condensates (Banani et al. 2017). The formation of these condensates is frequently driven by liquid–liquid phase separation (LLPS) (Shin and Brangwynne 2017). LLPS describes a biophysical process in which macromolecules self‐assemble and demix from the surrounding milieu to form distinct, concentrated phases, resembling liquid droplets (Figure 2). This non‐stoichiometric assembly is typically achieved by multivalent interactions involving intrinsically disordered protein regions (IDRs), structured domains, and nucleic acids (Kato et al. 2012; Li et al. 2012; Zhang et al. 2015). Biomolecular condensates dynamically rearrange their internal biomolecules and exchange components with the surrounding environment, providing a versatile platform for organizing biochemical reactions. Furthermore, condensates can exhibit properties ranging from liquid‐like to solid‐like states, influencing cellular function and pathology (Banani et al. 2017; Lyon et al. 2021; Sabari et al. 2020; Shin and Brangwynne 2017). Many nuclear bodies, or membraneless organelles (MLOs)—such as the nucleolus, nuclear speckles, histone locus bodies (HLBs), paraspeckles, and cleavage bodies—are now recognized as biomolecular condensates (Hirose et al. 2023). These nuclear biomolecular condensates are composed of diverse biomolecules and can reach micron‐scale dimensions. They often have complex internal organization and perform specialized functions similar to membrane‐bound organelles.

The role of biomolecular condensates in gene expression and pre‐mRNA processing has been characterized in the contexts of transcription and splicing, as reviewed recently (Giudice and Jiang 2024; Pei et al. 2024). These complex processes involve proteins enriched in IDRs, along with DNA and RNA, all possessing multivalency conducive to condensate formation. Condensates help explain complex gene regulation phenomena whose mechanisms were often unclear. For instance, diffraction‐sized transcriptional condensates enriched in Mediator and RNA pol II (> 300 nm) frequently assemble adjacent to super‐enhancers (Cho et al. 2018; Du et al. 2024; Kawasaki and Fukaya 2023). These condensates can facilitate robust gene activation and potentially bridge multiple enhancers and promoters indirectly over significant distances. Similarly, IDRs enriched in splicing and transcription factors are now known to drive condensate formation and selectively partition molecules into condensates, which can be modulated by post‐translational modifications (Guo et al. 2019; Lyons et al. 2023). Additionally, genome organization near nuclear speckles, which are enriched in splicing factors, correlates with enhanced splicing efficiency (Bhat et al. 2024). This proximity explains non‐linear increases in splicing efficiency with higher transcription levels (Ding and Elowitz 2019). Together, these findings support a condensate‐based model for understanding RNAPII mRNA transcription and pre‐mRNA processing (Giudice and Jiang 2024; Pei et al. 2024).

However, the role of biomolecular condensates in human pre‐mRNA 3′ end processing is less understood. Current models typically depict 3′ end processing factors assembling on RNA elements in fixed stoichiometries. APA is generally explained by linear models involving RNAPII elongation rate, competition between splicing and 3′ end processing, and *trans*‐acting factors binding near the PAS (Mitschka and Mayr 2022). Yet, accumulating evidence suggests that core 3′ end processing factors and regulators undergo phase separation and are localized into distinct nuclear biomolecular condensates. Recently, nuclear speckles were identified as a major hub for the 3′ end processing of poly(A) mRNAs (Yoon et al. 2025). This review synthesizes recent evidence on condensates in 3′ end processing, highlighting how IDRs within 3′ end processing factors and their regulators drive condensate formation and influence 3′ end formation. We also explore how distinct 3′ end processing mechanisms are functionally compartmentalized by nuclear biomolecular condensates. Lastly, we propose directions for future research aimed at elucidating the role of condensates in regulating 3′ end processing and other pre‐mRNA processing steps.

## 2 Biomolecular Condensates Formed by 3′ End Processing Factors: Formation and Function

In this section, we discuss the formation and function of biomolecular condensates involving 3′ end processing factors and their regulators. We focus on the role of IDRs in driving this process and review the emerging functions of biomolecular condensates in 3′ end processing.

### 2.1 Role of IDRs in Phase Separation of 3′ End Processing Factors

Condensate formation is frequently driven by LLPS mediated by IDRs and/or low‐complexity (LC) domains. IDRs and LC domains are particularly abundant in transcription factors, splicing factors, and RNA‐binding proteins, compared to the total proteome (Castello et al. 2012; Herzel et al. 2017; Liu, Perumal, et al. 2006). While the precise functions of many of these regions remain unclear, they frequently underlie condensate formation of transcription and splicing factors (Giudice and Jiang 2024; Pei et al. 2024). Because IDRs can provide site‐ or chemically specific multivalent interfaces, IDR‐containing proteins often act as “scaffolds” that are essential for condensate formation and maintenance (Banani et al. 2016; Holehouse and Alberti 2025). These scaffolds, in turn, can recruit low‐valency “client” proteins into the condensate (Banani et al. 2016). However, it is important to note that not all IDRs inherently drive phase separation, and that structured domains can also mediate this process (Holehouse and Alberti 2025).

**FIGURE 3:**
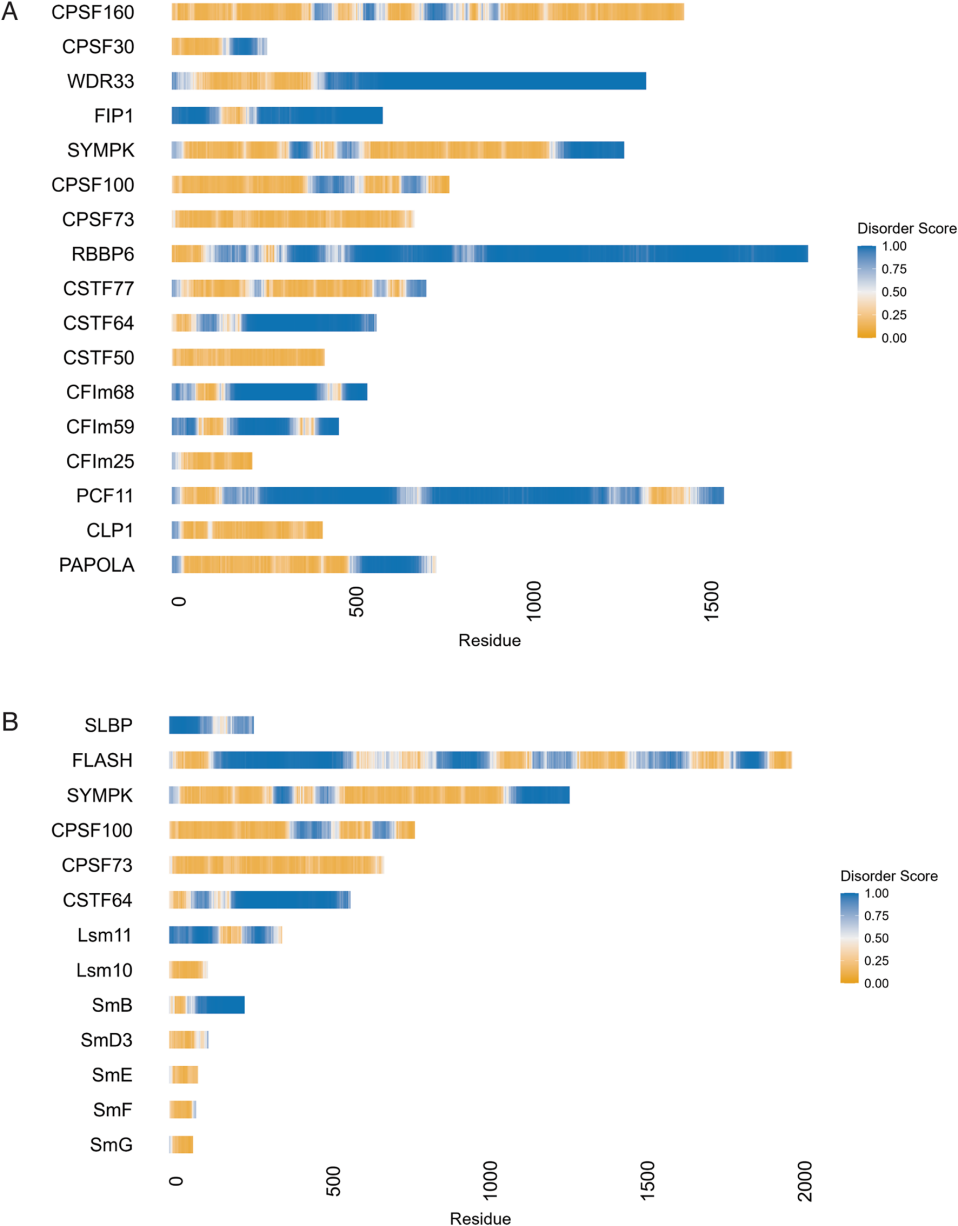
Core 3′ end processing factors for mRNAs are enriched in intrinsically disordered protein regions (IDRs). Predicted disorder scores for individual proteins were generated using AIUPred (Erdős and Dosztányi 2024), plotted across the protein sequences. A disorder score greater than 0.5 is considered disordered. Higher scores indicate greater predicted disorder. (A) Predicted disorder scores of core 3′ end processing factors for polyadenylated mRNAs. (B) Predicted disorder scores of core 3′ end processing factors for histone mRNAs.

Similar to transcription and splicing factors, IDRs are abundant in core 3′ end processing factors for both polyadenylated and histone mRNAs. Computational prediction suggests that substantial portions of these proteins are disordered (Figure 3). For instance, ~80% of the 1,792‐amino acid (aa) protein RBBP6 is predicted to be disordered. A similar pattern is observed in other core factors, including WDR33, CSTF64, FIP1, PCF11, CFIm59 (CPSF7), and CFIm68 (CPSF6), where well over 50% of their sequences are likely IDRs. Other factors like PAPOLA, SYMPLEKIN (SYMPK), and CPSF30 also contain extensive disordered segments. This pattern extends to core factors unique to histone 3′ end processing, such as SLBP, FLASH, SmB, and Lsm11 (Duronio and Marzluff 2017). However, structural and functional investigations have often neglected these IDRs, potentially due to experimental bias or difficulties in expressing and purifying these regions.

What are the functions of these IDRs in 3′ end processing? In vitro reconstitution studies indicate that some extensive IDRs in core factors are not strictly required for the catalytic steps of 3′ end cleavage. Notably, cleavage activity was achieved using both full‐length and truncated versions of 3′ end processing factors (Boreikaite et al. 2022; Schmidt et al. 2022). For example, truncated versions of RBBP6 (aa 1–253), WDR33 (aa 1–576), bovine PAP (aa 1–513), and PCF11 (aa 769–1555)—all lacking long N‐ or C‐terminal IDRs—were functional in these assays (Boreikaite et al. 2022). Whether the presence of these IDRs enhances cleavage efficiency has not been directly compared under identical assay conditions across studies. These observations suggest that long IDRs in several core 3′ end processing factors are dispensable for the cleavage reaction under the concentrated, simplified conditions in a test tube.

Recent studies suggest that several IDRs in core 3′ end processing factors drive condensate formation, both in vitro and in cells (Table 1). For example, PCF11 condensation depends on an α‐helical region within its N‐terminal IDR (Liu et al. 2025). The structured N‐terminal domain (NTD) of RBBP6 can independently phase separate, while its C‐terminal IDR promotes viscoelastic phase separation and influences localization within nuclear speckles (Yoon et al. 2025). The C‐terminal IDR of CFIm68, which contains an arginine/serine‐like domain (RSLD), also promotes condensate formation (Liu et al. 2023). Poly(A)‐binding protein nuclear 1(PABPN1), which binds the poly(A) tail (Keller et al. 2000; Wahle et al. 1991), undergoes LLPS driven by its N‐terminal IDR and RNA‐recognition motif (RRM) (Dai et al. 2022). CstF64 and CPSF30‐L undergo phase separation, likely facilitated by prion‐like low‐complexity domains (PLDs), although the role of these PLDs in phase separation has not yet been directly tested (Song et al. 2021; Wang, Choi, et al. 2018). This trend also extends to RNA‐binding proteins (RBPs) that directly regulate APA (summarized in Table 1), suggesting IDR‐mediated phase separation may be broadly important for 3′ end processing regulation. In the next section, we explore how these condensates might functionally influence 3′ end processing.

**TABLE 1:** Biomolecular condensates formed by pre‐mRNA 3′ end processing factors and RBP regulators.

Protein	Role in pre‐mRNA 3′ end processing (Reviewed in Boreikaite and Passmore 2023; Mitschka and Mayr 2022)	Biomolecular of condensate formation
RBBP6	Core 3′ end processing factor; endonuclease activation	Forms condensates in vitro and in cells. The full‐length protein exhibits viscoelastic properties. NTD can form liquid‐like droplets. Localizes to nuclear speckles mediated by its IDR subregion, which is important for the 3′ end processing of a large subset of genes (Yoon et al. 2025).
PCF11	Core 3′ end processing factor; binds RNAPII CTD and regulate transcription termination	Forms condensates in vitro and in cells, mediated by an alpha‐helical region within the IDR. Condensation facilitates RNAPII stalling (Liu et al. 2025)
CFIm68 (CPSF6)	Core 3′ end processing factor; binds FIP1 and enhance 3′ end processing	Forms condensates in vitro and in cells via the RSLD within its C‐terminal IDR. Condensation is regulated by CLK2 phosphorylation or osmotic stress, reduced in cancer, and influences APA (Jalihal et al. 2020; Liu et al. 2023)
CSTF64	Core 3′ end processing factor; RNA binding	Forms condensates in vitro. Contains PLD (Wang, Choi, et al. 2018).
CPSF30	Core 3′ end processing factor; RNA binding	Arabidopsis CPSF30‐L forms nuclear bodies with characteristics of LLPS (Song et al. 2021). Contains PLD.
PABPN1	Regulates APA by inhibiting binding of 3′ end processing factors	Forms condensates in vitro and in cells, mediated by both an N‐terminal IDR and RRM. Condensation is promoted by poly(A) RNAs and Quaking (QKI). Changes in condensation properties are linked to APA and diseases (Dai et al. 2022; Guan et al. 2023; Li et al. 2025).
TDP‐43	Regulates APA in a position‐dependent manner (Rot et al. 2017)	Forms condensates in vitro and in cells, mediated by the N‐terminal domain and a conserved region between C‐terminal IDRs. Condensation is important for binding and regulating the 3′ end processing of a subset of genes (Conicella et al. 2016; Hallegger et al. 2021; Molliex et al. 2015; Wang, Conicella, et al. 2018).
FUS	Regulates APA in a position‐dependent manner	Forms condensates in vitro and in cells, mediated by the N‐terminal PLD and the C‐terminal RNA‐binding domain. Condensation facilitates the recruitment of the RNAPII CTD (Burke et al. 2015; Murthy et al. 2019; Patel et al. 2015; Wang, Choi, et al. 2018).
hnRNP A3	Promotes intronic polyadenylation	Forms condensates in vitro (Wang, Choi, et al. 2018)
hnRNP E1 (PCBP1)	Enhances upstream PAS usage	Promotes DNA‐induced LLPS of cGAS and forms co‐condensates (Du and Chen 2018; Liao et al. 2021)
hnRNP I (PTBP1)	Binds the upstream element (USE) and promotes 3′ end processing	Forms condensates in vitro upon addition of RNA containing 3xUCUCU sequences (de Vries et al. 2024)
SRSF7	Binds upstream of proximal PAS and promotes proximal PAS usage	Binds to its own mRNA at a retained intron, inducing the formation of liquid‐like nuclear bodies (Königs et al. 2020)
U1 snRNP	Inhibits intronic polyadenylation through telescripting	U1 snRNP protein component U1‐70K forms condensates in vitro and in cells, mediated by LC domains (Hu et al. 2022; Xue et al. 2019; Zhou et al. 2025)
CELF2	Regulates APA by competing with 3′ end processing factors	Forms condensates in vitro and in cells, mediated by its IDR (Li et al. 2024)
CPEB1	Promotes the usage of proximal PAS	Forms condensates in cells, requiring RNA‐binding domains (Duran‐Arqué et al. 2022)
CPEB4	Promotes the usage of proximal PAS	Forms condensates in vitro and in cells, mediated by the N‐terminal domain containing an IDR (Duran‐Arqué et al. 2022; Guillén‐Boixet et al. 2016; Lin et al. 2010)
ELAVL1 (HuR)	Promotes the usage of distal PAS	Forms mRNA‐rich, liquid‐like condensates when artificially fused to microtubules (Maucuer et al. 2018)
CIRBP	Binds to the 3′ UTR near the PAS and regulates APA	Forms condensates in vitro, mediated by the RG/RGG region (Bourgeois et al. 2020; Lenard et al. 2021)
FCA	Regulates APA of crucial genes in development (Koornneef et al. 1991; Sheldon et al. 1999)	Forms condensates in vitro and in cells, mediated by the PLD (Fang et al. 2019)
RBM10	Regulates APA by interacting with Star‐PAP (Mohan et al. 2018)	A mutation in the RRM domain, implicated in colon cancer, leads to increased condensation of RBM10 in cells (Banani et al. 2022)

### 2.2 Functions of Condensates Formed by 3′ End Processing Factors

The roles of biomolecular condensates formed by 3′ end processing factors are just beginning to be understood. It has been widely recognized that molecular crowding reagents, such as polyethylene glycol (PEG) or polyvinyl alcohol (PVA), are either required for or markedly enhance the pre‐mRNA cleavage efficiency of in vitro assays using recombinant 3′ end processing factors or nuclear extracts (Huang, Liu, et al. 2023; McLauchlan et al. 1988; Zarkower et al. 1986). This enhancement has been attributed to molecular crowding effects, which simply increase the local concentration of proteins and RNAs, thereby facilitating the reaction. However, these crowding reagents may also induce phase separation (Alberti et al. 2019), selectively partitioning specific components into condensates. Such phase separation could thus facilitate pre‐mRNA processing not only by concentrating essential biomolecules but also by excluding unrelated or inhibitory factors. Moreover, it may alter the RNA‐binding affinities or biophysical properties of the 3′ end processing factors themselves. However, whether this is the case in the cellular environment remains to be elucidated, though several examples support this possibility as described below.

**FIGURE 4:**
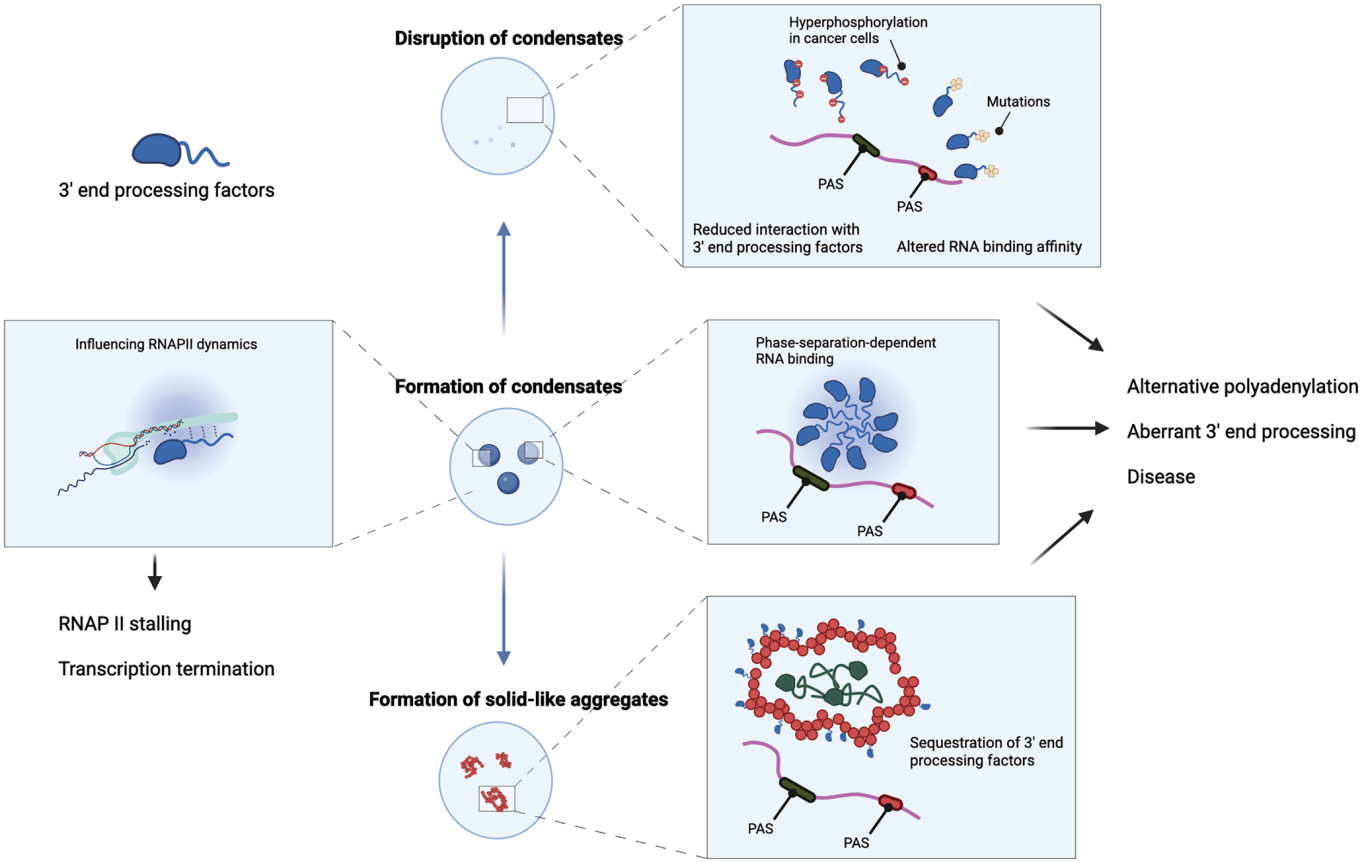
Functional roles of bimolecular condensates formed by 3′ end processing factors. Phase separation of 3′ end processing factors enables their condensation‐dependent binding to specific RNA sequences and influences RNAPII dynamics; thereby, modulating APA and transcription termination. Disruption of condensate formation or aggregation can misregulate APA and contribute to pathologies such as cancer. Specific examples are provided in Section 2.2.

Condensation of 3′ end processing factors can modulate APA (Figure 4). TAR DNA‐binding protein 43 (TDP‐43), an RBP that regulates APA in a position‐dependent manner (Rot et al. 2017), has well‐characterized phase separation properties (Conicella et al. 2016; Molliex et al. 2015; Rot et al. 2017; Wang, Choi, et al. 2018). Notably, the condensation of TDP‐43 is essential for binding a subset of transcripts featuring specific motif patterns (Hallegger et al. 2021). Point mutations that disrupt TDP‐43 condensation to varying degrees specifically impair 3′ end processing of these target transcripts, primarily by reducing RNA‐binding affinity. Thus, condensation can tune the RNA‐binding affinity or specificity of 3′ end processing factors, potentially influencing APA by altering target selection. Consequently, cellular mechanisms that regulate condensation may also function as important regulatory pathways controlling APA. CFIm68 exemplifies this concept, as its condensation is regulated by phosphorylation and osmotic stress. CFIm68 acts as a position‐specific activator of PAS usage (Gruber et al. 2012; Hwang et al. 2016; Martin et al. 2012; Zhu et al. 2018). CLK2‐mediated phosphorylation controls CFIm68 phase separation; mutants unable to form condensates cause opposite APA shifts compared to the wild type (Liu et al. 2023). Additionally, osmotic stress triggers CFIm68 condensation and impairs 3′ end processing (Jalihal et al. 2020). Because CFIm68 interacts with Fip1 of the mPSF complex (Figure 1), its phase separation may recruit additional core 3′ end processing factors into condensates, thereby redirecting their RNA‐target interactions or sequestering the processing complex itself from the chromatin. It remains an open question whether the IDRs of other 3′ end processing factors promote selective co‐condensation among each other or across distinct nuclear subcompartments.

Similarly, dysregulation of condensation is linked to pathological APA (Figure 4). In cancer cells, a reduced number of CFIm68 condensates is associated with 3′ UTR shortening (Liu et al. 2023)—a phenomenon frequently observed in cancer cells (Mayr and Bartel 2009). PABPN1 undergoes LLPS that can be further promoted by Quaking (QKI), an RBP with tumor suppressor activity (Li et al. 2025). Notably, reduced QKI expression in colorectal cancer cells attenuates PABPN1 LLPS, correlating with a proximal shift in APA and increased cell proliferation. In oculopharyngeal muscular dystrophy (OPMD), an alanine expansion mutation in PABPN1 converts liquid‐like condensates to solid‐like aggregates, sequestering CFIm25 and causing aberrant APA (Guan et al. 2023). Together, these examples show that condensation of 3′ end processing factors plays an important role in regulating APA and is often disrupted in disease.

Condensation can also influence RNAPII dynamics, thereby affecting 3′ end processing and transcription termination (Figure 4). PCF11, an essential 3′ end processing factor, binds the RNAPII C‐terminal domain (CTD) and also regulates transcription termination (Kamieniarz‐Gdula et al. 2019; Zhang et al. 2005). Recent work indicates that phosphorylation at Threonine 4 (Thr4‐P) of the RNAPII CTD promotes PCF11 condensation, which may mechanically slow down and stall RNAPII to facilitate transcription termination (Liu et al. 2025). This model is consistent with previous observations that condensates can exert mechanical forces, such as pulling, on associated molecules (Chappidi et al. 2024). Since RNAPII elongation rate and PAS selection are kinetically coupled (Elkon et al. 2013), PCF11 condensation likely influences APA indirectly. Similarly, FUS regulates APA by inducing RNAPII stalling and recruiting core 3′ end processing factors (Masuda et al. 2015, 2020). Given that FUS condensates recruit RNAPII CTD (Burke et al. 2015), they might function analogously to PCF11 condensates. This raises an intriguing question: can the phosphorylation status of RNAPII CTD determine its selective partitioning into 3′ end processing condensates? For example, Serine 2 phosphorylation (Ser2‐P) of the RNAPII CTD promotes its selective partitioning into splicing condensates over transcriptional condensates (Guo et al. 2019). Thr4‐P, which is enriched near gene ends (Kamieniarz‐Gdula et al. 2019), might similarly facilitate switching from splicing condensates to those involved in 3′ end processing. Alternatively, given the role of Ser2‐P in coupling splicing with 3′ end processing (Ahn et al. 2004), factors related to both steps might partition into shared condensates. This will be the subject of future study.

In summary, similar to transcription and splicing factors (Giudice and Jiang 2024; Pei et al. 2024), IDRs in 3′ end processing factors significantly contribute to phase separation. Phase separation likely confers emergent properties that contribute to regulating 3′ end processing, such as altering RNA‐binding affinity or biophysical properties, and promoting selective partitioning of biomolecules. Crucially, dysregulated phase separation is implicated in disease‐related APA. However, it remains important to acknowledge the challenge of determining whether an observed phenotype is caused solely by altered condensation. Mutations used to disrupt phase separation can also affect other molecular functions. Since the study of condensates in pre‐mRNA processing is still new and rapidly developing, progress will depend on careful experiments and the development of new tools and ideas.

## 3 Compartmentalization of Pre‐mRNA 3′ End Processing by Nuclear Biomolecular Condensates

While many 3′ end processing factors can self‐assemble into small, transient condensates (see Section 2), some of them are also incorporated into larger, more stable nuclear biomolecular condensates—commonly known as MLOs or nuclear bodies. These structures were first described in the early 19th century, long before the biophysical principle underlying their formation was understood (Valentin 1836; Wagner 1835). They are now understood to be biomolecular condensates, many of which exhibit liquid‐like properties (Courchaine et al. 2016; Faber et al. 2022; Hirose et al. 2023; Lafontaine et al. 2021). These condensates are typically of mesoscopic size (0.2–2 μm) and possess significant compositional complexity (Banani et al. 2017; Hirose et al. 2023). Nuclear biomolecular condensates are thought to function as specialized hubs for gene expression, located close to relevant genomic regions. For example, nucleoli, Cajal bodies, nuclear speckles, and HLBs are situated near genomic regions containing ribosomal DNA (rDNA), small nuclear RNA (snRNA) genes, highly expressed genes encoding poly(A) mRNAs, and histone gene clusters, respectively (Faber et al. 2022; Ilik and Aktas 2022; Lafontaine et al. 2021; Nizami et al. 2010). Yet, precisely defining the functions of these biomolecular condensates remains challenging. One reason is that many of their resident factors are also found distributed throughout the nucleoplasm, where they can function independently (Chaturvedi and Belmont 2024). This complicates the interpretation of experimental phenotypes, and thus much of the evidence linking nuclear biomolecular condensates to specific functions remains correlative. In this section, we review the association of 3′ end processing factors with nuclear biomolecular condensates and discuss how compartmentalization facilitates distinct 3′ end processing events, contributing to efficient gene expression.

### 3.1 Pre‐mRNA 3′ End Processing Factors Associated With Nuclear Biomolecular Condensates

3′ end processing of pre‐mRNAs requires precise spatial and temporal regulation for both poly(A) and histone mRNAs. Most 3′ end processing occurs co‐transcriptionally and is often coupled with splicing for intron‐containing genes (Boreikaite and Passmore 2023). Therefore, this process must be closely coordinated with transcription and splicing machinery. Additionally, while the 3′ end processing machineries for poly(A) and histone mRNAs share a subset of core factors (CPSF73, CPSF100, SYMPLEKIN, and CstF64), each pathway also includes unique components, likely requiring distinct regulatory environments. Histone genes represent only a small fraction of protein‐coding genes (~72 genes in humans) and are tightly clustered in several genomic loci (Duronio and Marzluff 2017). Histone proteins, however, are among the most abundant cellular proteins, reflecting a high demand for their production (Cooper 2000). Thus, compartmentalizing these reactions is likely important for precisely controlling distinct 3′ end processing events.

**FIGURE 5:**
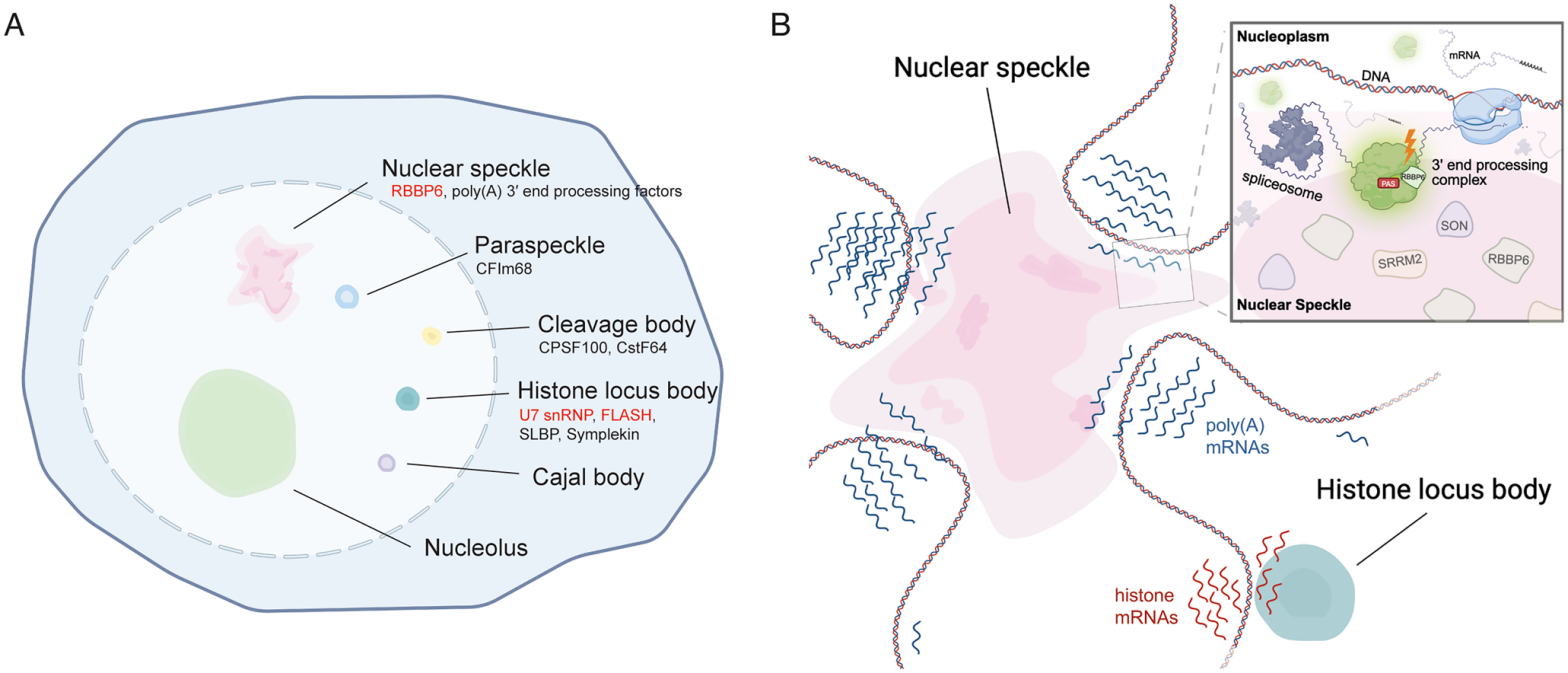
Nuclear biomolecular condensates compartmentalize distinct 3′ end processing pathways. (A) Examples of nuclear biomolecular condensates (or membraneless organelles). Associated 3′ end processing factors are indicated, with factors specifically enriched in each organelle highlighted in red. (B) Nuclear speckles and histone locus bodies compartmentalize the 3′ end processing of polyadenylated and histone mRNAs, respectively. Nuclear speckles function as central hubs for polyadenylated mRNA processing, containing splicing factors and core poly(A) 3′ end processing factors, with specific enrichment of RBBP6. Highly expressed genes and transcriptionally active, gene‐rich chromosomal regions localize near nuclear speckles. This enables efficient coordination among transcription, splicing, and 3′ end processing. In contrast, histone locus bodies are enriched in histone mRNA processing factors, including those required for histone mRNA 3′ end formation. They associate closely with histone gene clusters, ensuring efficient histone mRNA expression specifically during S phase.

Indeed, core 3′ end processing factors exhibit distinct associations with nuclear biomolecular condensates (Figure 5A). Imaging analysis suggests that RBBP6 is specifically enriched in nuclear speckles, whereas most other poly(A) processing factors are broadly distributed throughout the nucleoplasm (Yoon et al. 2025). Nonetheless, proteomics studies indicate that almost all core poly(A) 3′ end processing factors interact with nuclear speckle proteins or RNAs (Dopie et al. 2020; Saitoh et al. 2004; Wu, Ye, et al. 2024). This suggests that most factors involved in poly(A) mRNA processing associate transiently or peripherally with nuclear speckles, while RBBP6 represents an integral component. Similarly, several histone 3′ end processing factors, such as FLASH, U7 snRNP, and SLBP, show enrichment in HLBs (Geisler et al. 2023; Liu, Murphy, et al. 2006). Among factors shared between the poly(A) and histone mRNA 3′ end processing pathways, only SYMPLEKIN is enriched within HLBs during S phase (Tatomer et al. 2014; Wagner et al. 2007).

In addition to nuclear speckles and HLBs, several core 3′ end processing factors are associated with cleavage bodies and paraspeckles. CPSF100 and CstF64 are enriched in cleavage bodies, which typically appear as one to four foci per nucleus (Schul et al. 1996). Cleavage bodies exhibit cell cycle‐dependent dynamics and are frequently found adjacent to HLBs during S phase. However, their function is unclear, and whether they contain RNA remains debated (Li et al. 2006; Schul et al. 1996). All three components of the CFIm complex (CFIm25, CFIm59, and CFIm68) have been detected in paraspeckles (Dettwiler et al. 2004; Naganuma et al. 2012). CFIm68 is partially enriched in paraspeckles under basal conditions (Cardinale et al. 2007) but forms condensates upon HIV‐1 infection and relocates to nuclear speckles with HIV‐1 replication complexes; these processes are important for HIV‐1 nuclear reverse transcription and productive infection (Francis et al. 2020; Selyutina et al. 2020; Tomasini et al. 2024).

In summary, core 3′ end processing factors associate with nuclear biomolecular condensates to varying degrees: (1) specific enrichment (e.g., RBBP6 in nuclear speckles; FLASH and U7 snRNP in HLBs), (2) partial enrichment (e.g., SLBP and SYMPLEKIN in HLBs; CPSF100 and CstF64 in cleavage bodies; CFIm68 in paraspeckles), and (3) broad nucleoplasmic distribution with detectable interactions via proteomics (e.g., most poly(A) mRNA processing factors with nuclear speckles). In the next section, we discuss how this distribution supports functional compartmentalization of different 3′ end processing events, focusing primarily on nuclear speckles and HLBs.

### 3.2 Nuclear Speckles as Major Hubs for 3′ End Processing of Polyadenylated mRNAs

Recent studies have highlighted the role of nuclear speckles as key hubs for gene expression, coordinating RNAPII transcription, splicing, and 3′ end processing (Chaturvedi and Belmont 2024; Faber et al. 2022; Ilik and Aktas 2022). Nuclear speckles typically appear as 20–50 irregularly shaped foci, ranging from 0.3 to 3 μm in size (Hirose et al. 2023; Spector and Lamond 2011). They contain hundreds of proteins, including many splicing factors, non‐coding RNAs, and poly(A) RNAs (Dopie et al. 2020; Huang and Spector 1992; Saitoh et al. 2004; Wu, Xiao, et al. 2024; Wu, Ye, et al. 2024). Nuclear speckles exhibit a multi‐layered organization (Fei et al. 2017) and display viscoelastic properties (Yoon et al. 2025; Zhang et al. 2024, 2025). The “gene expression hub” model is based on several observations: (1) Highly expressed genes and active gene‐dense chromosomal regions are situated near nuclear speckles (Chen et al. 2018; Quinodoz et al. 2018, 2021; Takei et al. 2025; Zhang et al. 2021); (2) gene activation, such as during heat shock or p53 induction, is accompanied by the relocation of target genes toward nuclear speckles (Alexander et al. 2021; Khanna et al. 2014); (3) Splicing efficiency is highest near nuclear speckles, and artificially tethering pre‐mRNAs to nuclear speckles enhances splicing (Bhat et al. 2024); (4) 3′ end processing of a large subset of genes occurs near nuclear speckles (Yoon et al. 2025). An alternative, though not mutually exclusive, model proposes that nuclear speckles primarily serve as storage sites for splicing factors (Matta 1994; Spector and Lamond 2011).

Although research has historically emphasized nuclear speckles' roles in gene expression or splicing, a recent study suggests that they are also involved in 3′ end processing of poly(A) mRNAs (Yoon et al. 2025). This conclusion is based on several key observations. First, RBBP6, an essential 3′ end processing factor, is specifically enriched within nuclear speckles. This localization is mediated by its C‐terminal IDR and is crucial for the 3′ end processing of a large subset of genes. For instance, only RBBP6 variants containing both the 3′ end processing activation domain and the IDR subregion required for nuclear speckle localization rescue 3′ end processing defects caused by RBBP6 depletion. Second, disrupting nuclear speckle scaffold proteins, such as SRRM2 and SON (Ilik et al. 2020), impairs 3′ end processing and transcription termination. Third, more than 50% of uncleaved pre‐mRNAs are specifically enriched near nuclear speckles. These genes tend to have higher GC content, consistent with their known association with nuclear speckles (Barutcu et al. 2022; Chen et al. 2018; Takei et al. 2025; Wu, Xiao, et al. 2024). Collectively, the enrichment of substrates (uncleaved pre‐mRNAs), essential processing factors (RBBP6 and transiently associated factors), and products (poly(A) RNAs) strongly suggest that nuclear speckles serve as major hubs for 3′ end processing. The specific enrichment of RBBP6 in nuclear speckles may reflect its very low cellular abundance—the lowest among all core 3′ end processing factors (Huang, Szklarczyk, et al. 2023). Thus, nuclear speckles could provide the necessary concentration of RBBP6 at active processing sites. This scenario parallels HLBs, where FLASH and U7 snRNP—also expressed at low levels—are selectively concentrated to enable efficient histone pre‐mRNA processing (Duronio and Marzluff 2017). Together, these findings further support the role of nuclear speckles as gene expression hubs coordinating transcription, splicing, and 3′ end processing (Figure 5B).

Given their structural and evolutionary similarities, RBBP6, SRRM2, and SON may have co‐evolved to establish nuclear speckles as hubs for coordinating pre‐mRNA processing. SRRM2 and SON are essential for nuclear speckle formation and widely used as nuclear speckle markers (Ilik et al. 2020). Notably, all three proteins underwent dramatic length expansion during evolution, primarily through the acquisition of extensive IDRs (Ilik and Aktas 2022). In contrast, the yeast homologs of RBBP6 and SRRM2, which function in 3′ end processing and splicing respectively, lack these long IDRs (Hill et al. 2019; Jia and Sun 2018), and yeast also lack nuclear speckles. The IDRs of SRRM2 and SON drive nuclear speckle formation through phase separation, while the IDR of RBBP6 mediates its localization to nuclear speckles and likely facilitates protein interactions within these nuclear bodies (Dion et al. 2024; Xu et al. 2022; Yoon et al. 2025; Zhang et al. 2024). Collectively, these findings support an evolutionary scenario in which the acquisition of extended IDRs in these proteins contributed to the formation of nuclear speckles, spatially integrating separate pre‐mRNA processing steps.

An important question is how frequently RNAPII‐dependent gene expression processes occur near nuclear speckles. Analyses suggest approximately half of 3′ end processing events occur in proximity to nuclear speckles (Yoon et al. 2025). Genome‐wide studies confirm a strong correlation between speckle proximity and highly expressed genes or active genomic compartments, although not all actively transcribed genes associate with nuclear speckles (Chen et al. 2018; Quinodoz et al. 2018, 2021; Zhang et al. 2021). Imaging studies report low to moderate levels (up to ~45%) of colocalization between nuclear speckles and active chromatin marks, transcriptional condensates, or nascent RNAs (Du et al. 2024; Guo et al. 2019; Shah et al. 2018; Takei et al. 2021, 2025; Wei et al. 1999). Genomic associations with speckles are generally conserved across cell types (Takei et al. 2025; Zhang et al. 2021), with lower speckle association for long, low‐GC‐content, or cell‐type‐specific genes (Takei et al. 2025). These results suggest that nuclear speckles primarily support gene expression from densely packed, highly expressed genomic regions.

However, several caveats complicate these interpretations. First, most imaging analyses are limited by diffraction‐limited resolution (~200–300 nm), potentially missing smaller condensates or subtle peripheral structures (Takei et al. 2025). Marker selection and staining methods also influence observations. Second, interactions between genomic DNA or transcriptional condensates and nuclear speckles are transient (Alexander et al. 2021; Cho et al. 2018; Khanna et al. 2014; Quinodoz et al. 2018), complicating accurate quantification. Third, even moderate spatial separation between transcriptional condensates and nuclear speckles may allow functional coupling via extended pre‐mRNA molecules, which can span 40–100 nm or more (Gopal et al. 2012; Lubeck and Cai 2012; Paul et al. 2024). Transcription regulation requires specificity in both locus and condensate composition (Pei et al. 2024), whereas constitutive splicing and 3′ end processing can occur in more general processing hubs like nuclear speckles. Thus, functional coupling between genes or transcriptional condensates and nuclear speckles may occur even without direct overlap. Surprisingly, super‐resolution imaging revealed that transcriptional condensates are situated at the periphery of nuclear speckles (Park et al. 2022), further supporting this hypothesis. Together, these caveats suggest that the actual fraction of gene expression events associated with nuclear speckles might be significantly underestimated.

### 3.3 Histone Locus Body Mediates 3′ End Processing of Replication‐Dependent Histone mRNAs

Histone proteins are among the most abundant cellular proteins and serve as the primary structural components of chromatin (Cooper 2000; Jiang and Berger 2023). Thus, large amounts of histone mRNAs must be synthesized during S phase under precise regulatory control. Histone genes are densely clustered in several genomic loci, often with very little intergenic space (Geisler et al. 2023; Marzluff et al. 2008). This genomic arrangement necessitates accurate and efficient 3′ end processing and transcription termination. For comparison, during poly(A) mRNA processing, RNAP II typically transcribes hundreds to thousands of nucleotides downstream of the cleavage site (Proudfoot 1989, 2016). Such extended transcription could cause interference when genes are closely spaced, as with histone genes. Indeed, metazoan histone mRNAs undergo a unique 3′ end processing mechanism involving a single endonucleolytic cleavage step (Dominski and Marzluff 2007). This highly sequence‐specific cleavage occurs between a conserved stem‐loop structure and a HDE. These elements are recognized by SLBP and U7 snRNA (part of U7 snRNP), respectively. SLBP levels increase in late G1, aligning with histone gene transcription during S phase (Whitfield et al. 2000). The cleavage reaction is carried out by a complex comprising U7 snRNP (including the Sm ring, Lsm10, and Lsm11), FLASH, SLBP, and the histone cleavage complex (HCC) (Skrajna et al. 2018; Sun et al. 2020; Yang et al. 2013). Notably, the HCC shares core subunits with the poly(A) mRNA 3′ end processing machinery, such as CPSF73, CPSF100, SYMPLEKIN, and CstF64 (Dominski et al. 2005; Kolev and Steitz 2005; Mandel et al. 2006; Yang et al. 2013).

HLBs are nuclear biomolecular condensates that form via phase separation at replication‐dependent histone gene loci in animal cells (Hur et al. 2020). These structures are enriched in histone mRNA processing factors (Duronio and Marzluff 2017; Geisler et al. 2023). Concentrating these factors within HLBs is critical for histone gene expression (Tatomer et al. 2016; Wagner et al. 2007). For instance, a high local concentration of U7 snRNP within HLBs is crucial for efficient processing (Wagner et al. 2007). Additionally, the protein NPAT is necessary for both HLB formation and histone gene expression (Ma et al. 2000; White et al. 2011; Zhao et al. 2000). HLBs are dynamically regulated throughout the cell cycle, particularly in size (Hur et al. 2020). Among the 3′ end processing factors shared between histone and poly(A) pathways, only SYMPLEKIN is specifically enriched in HLBs during S phase (Tatomer et al. 2014). In contrast, CPSF100 and CstF64 are enriched in separate nuclear biomolecular condensates called cleavage bodies, typically present in one to four foci per nucleus (Schul et al. 1996). An interesting observation is that cleavage bodies are also dynamically regulated during the cell cycle and are found adjacent to HLBs during S phase (Schul et al. 1999). Thus, cleavage bodies might support histone 3′ end processing by supplying concentrated CPSF100 and CstF64 near HLBs. Because CstF64 is mostly disordered (Figure 3) and can undergo phase separation (Table 1), it may also serve as a scaffold for cleavage bodies. Nevertheless, their precise formation mechanisms and functional roles remain to be elucidated.

Phase‐separated HLBs likely compartmentalize distinct 3′ end processing machineries for efficient and histone‐specific pre‐mRNA processing. HLBs increase local concentrations of low‐abundance factors, such as U7 snRNP and FLASH, facilitating efficient processing (Duronio and Marzluff 2017). This is analogous to the enrichment of low‐abundance RBBP6 in nuclear speckles for poly(A) mRNA processing (Yoon et al. 2025). In addition, phase separation may ensure processing specificity by excluding canonical poly(A) 3′ end processing factors not needed for histone mRNA maturation. Notably, disruptions in SLBP, U7 snRNP, or FLASH result in defective histone mRNA processing and transcriptional readthrough (Godfrey et al. 2006; Lanzotti et al. 2002; Tatomer et al. 2016; Wagner et al. 2007). Such failures often activate cryptic downstream PAS, resulting in the generation of polyadenylated histone mRNAs. This suggests that poly(A) processing factors are recruited as a backup mechanism and/or that the loss of phase separation allows aberrant interactions between the HCC and the poly(A) machinery. Interestingly, CPSF73 acts as the endonuclease for both histone and poly(A) mRNAs but requires distinct proteins for its activation: Lsm10 (in U7 snRNP) for histone processing and RBBP6 for poly(A) processing (Boreikaite et al. 2022; Hill et al. 2019; Schmidt et al. 2022; Sun et al. 2020). These cofactors may activate CPSF73 through mechanisms specific to each processing pathway. Therefore, selective recruitment of U7 snRNP versus RBBP6 may be critical for proper CPSF73 activation. The existence of separate HLBs and nuclear speckles, enriched respectively with U7 snRNP and RBBP6, likely promotes this functional separation (Figure 5B).

## 4 Conclusions

In this review, we have explored the emerging roles of biomolecular condensates in pre‐mRNA 3′ end processing. Notably, many proteins involved in 3′ end processing contain IDRs and undergo phase separation. This phenomenon can regulate 3′ end processing through mechanisms that extend beyond conventional protein–protein or protein‐RNA interactions characterized by fixed stoichiometry. Phase separation also drives the formation of nuclear biomolecular condensates, establishing specialized nuclear subcompartments that are spatially coordinated with the broader three‐dimensional genome architecture. Such compartmentalization enhances processing efficiency by concentrating essential factors and promotes spatial segregation of distinct molecular pathways, such as 3′ end processing of canonical polyadenylated mRNAs and replication‐dependent histone mRNAs.

Since the role of biomolecular condensates in pre‐mRNA 3′ end processing is only beginning to be understood, many exciting questions remain. In this respect, 3′ end processing offers a unique experimental system for exploring fundamental aspects of biomolecular condensates and its role in RNA processing. A major advantage is that both poly(A) and histone mRNA 3′ end processing reactions have been biochemically reconstituted in vitro (Boreikaite et al. 2022; Schmidt et al. 2022; Sun et al. 2020). These require only 13–14 proteins and an RNA substrate, which is much simpler than the human transcription or splicing machinery. Importantly, many 3′ end processing factors contain IDR and undergo phase separation. The reconstituted reactions provide quantifiable readouts, such as cleavage and/or polyadenylation efficiency, allowing direct assessment of the functional roles of phase separation. Additionally, individual protein factors and RNA substrate sequences can be readily modified, and other regulatory components can be introduced, offering flexibility and experimental control. Thus, these in vitro systems represent valuable tools for studying how organization into a condensate affects biochemical reactions such as RNA processing. Finally, insights gained from these biochemical approaches can be validated in cells using advanced imaging and high‐throughput methods capable of globally quantifying 3′ end processing events (Shepard et al. 2011; Yoon et al. 2021).

Several outstanding questions remain to be addressed in future studies. First, does phase separation influence 3′ end processing efficiency or substrate RNA recognition? It is currently unclear whether condensates form constitutively during 3′ end processing or selectively assemble on specific transcripts. Such condensates might facilitate the 3′ end processing reaction and could influence RNA‐binding specificity. For instance, phase separation might regulate the choice of alternative PASs (APA) or enable recognition of non‐canonical PASs, which often lack consensus motifs but are actively utilized in vivo. Second, does phase separation facilitate coupling between 3′ end processing and other condensates involved in transcription or splicing, possibly mediated by phosphorylation states of the RNAPII CTD (Guo et al. 2019)? Finally, important questions remain regarding the broader role of nuclear biomolecular condensates in regulating 3′ end processing. For example, what mechanisms organize the genome around these condensates? Could gene‐specific APA or alternative splicing be regulated by dynamic changes in gene proximity to nuclear speckles? Addressing these questions will significantly enhance our understanding of 3′ end processing and how it is integrated with broader gene expression control mechanisms involving biomolecular condensates.

## Author Contributions

**Yoseop Yoon:** conceptualization (lead), investigation (lead), methodology (lead), writing – original draft (lead), writing – review and editing (lead). **Liang Liu:** investigation (supporting), methodology (supporting). **Cailyx Quan:** methodology (supporting), resources (supporting). **Yongsheng Shi:** conceptualization (lead), funding acquisition (lead), investigation (lead), methodology (lead), writing – review and editing (supporting).

## Conflicts of Interest

The authors declare no conflicts of interest.

## Related WIREs Articles

Hitting the mark: Localization of mRNA and biomolecular condensates in health and disease

## Data Availability

Data sharing is not applicable to this article as no new data were created or analyzed in this study.
